# Ag NPs-Assisted Synthesis of Stable Cu NPs on PET Fabrics for Antibacterial and Electromagnetic Shielding Performance

**DOI:** 10.3390/polym12040783

**Published:** 2020-04-02

**Authors:** Ke Wang, Qian Ma, Yuanming Zhang, Shudong Wang, Guangting Han

**Affiliations:** 1 State Key Laboratory of Bio-Fibers and Eco-Textiles, College of Textile & Clothing, Qingdao University, Qingdao 266071, China; ycfywk@126.com; 2 Jiangsu Research and Development Center of the Ecological Textile Engineering and Technology, Department of Textile and Clothing, Yancheng Polytechnic College, Yancheng 224005, China; ycfymq@126.com; 3 Department of Mechanical Engineering, North Dakota State University, Fargo, ND 58108, USA; 4 National Engineering Laboratory for Modern Silk, College of Textile and Clothing Engineering, Soochow University, Suzhou 215123, China

**Keywords:** Cu NPs, Ag NPs, polydopamine, PET fabrics, antibacterial, electromagnetic interference shielding

## Abstract

In this study, Cu/Ag/polydopamine (PDA)/polyester (PET) fabrics were fabricated for multi-functional textiles. The PET fabrics were firstly modified by dopamine to form a polydopamine (PDA) layer on the fiber surface, then Ag nanoparticles (Ag NPs) were anchored on fiber surface through chelation between PDA and Ag^+^ ions, and the Ag NPs were further used as catalytic seeds for in situ reduction of Cu nanoparticles (Cu NPs). The surface morphology, chemistry, and crystalline structure of the prepared PET fabrics were characterized by scanning electron microscopy (SEM), energy dispersive X-ray spectroscopy (EDS), and X-ray diffraction (XRD). As expected, Cu NPs were evenly dispersed on the surface of fibers. The Cu/Ag/PDA/PET fabrics showed good antibacterial property against *Escherichia coli* and exhibited excellent electromagnetic interference (EMI) shielding ability. The Cu/Ag/PDA/PET fabrics with high performance antibacterial and EMI shielding properties can be applied as functional protective textiles.

## 1. Introduction

Surface metallization endows textiles with a variety of functions, such as antibacterial, anti-ultraviolet, conductive, and electromagnetic shielding performance [1,2,3,4]. Chemical plating is a commonly adopted approach to metallize textiles, and the process of which includes desizing, roughening, sensitization, activation, and deposition [5,6,7]. For example, stannous chloride (SnCl_2_) is generally used to form gel on fabric surface to reduce the activator palladium chloride (PdCl_2_), the noble metal catalytic is formed on the surface of the fabric, the activation energy of metal deposition during chemical plating process is reduced, leading to increased metal deposition rate. Traditional chemical plating process is complicated, and the involved SnCl_2_ and PdCl_2_ can cause environmental pollution due to their toxic nature [8,9]. Moreover, the loose coating with weak bonding between metal and fabric restricts its practical applications. It is still challenging to develop a simple method of chemical metal plating on fabric surface with high fastness.

Mussel-inspired PDA coating has become one of the most important surface modification methods. Due to the simple experimental strategy, room temperature reaction, adhesiveness, and universality, PDA has been widely applied to almost all kinds of materials [10,11,12,13]. More importantly, PDA coatings contain many functional groups (such as catechol, amine, and imine), enabling PDA to perform as a versatile platform for secondary reactions to generate desirable functions [14,15]. Mussel-inspired PDA coating has attracted great attention in textiles. Lu et al. prepared silver nanoparticles on polydopamine coated silk fibers for antibacterial application [16]. Ran et al. deposited ZnO nanoparticles on cotton fabrics through polydopamine templates for UV protection [17]. Xu et al. deposited Ag nanoparticles on polydopamine-templated cotton fabrics for oil/water separation and self-cleaning [18]. Yang et al. grew Cu nanoparticles on polydopamine modified cotton fabrics for superhydrophobicity and antibacterial activities [19]. The surface of PDA-coated fabrics contains catechol groups that can help form nanoparticles from metal ions through chelate reaction. However, the reaction often takes a long time and the efficiency is too low for commercialization.

Herein, we report a simple but effective method to deposit Cu NPs on Polyester (PET) fabrics through in situ reduction. The PET fabrics were firstly templated with a PDA layer on the surface of fibers. The catechol groups of PDA chelated with Ag^+^ ions to form Ag NPs, which were further used as catalytic seeds for in situ reduction of Cu NPs. The PDA also has high adhesiveness to further immobilize Cu NPs on the surface of fibers. The morphology and structure of the Cu NPs-coated PET fabrics were characterized by scanning electron microscopy-energy dispersive X-ray spectroscopy (SEM-EDS) and X-ray diffraction (XRD). Moreover, the wettability, antibacterial and electromagnetic shielding performance of the coated PET fabrics were investigated.

## 2. Materials and Methods

### 2.1. Materials

Polyester fabrics (PET, plain weave, 24 g/m^2^ mass, density 810/10 cm warp and 630/10 cm weft, cleaned by acetone and deionized water before use), was purchased from Suzhou Gaoyan Textile Technology Co., Ltd., Suzhou, China. 3-hydroxytyramine hydrochloride (dopamine hydrochloride), silver nitrate (AgNO_3_), copper chloride (CuCl_2_), ethylene diamine tetraacetic acid (EDTA), dimethylamine borane (DMAB) and boric acid (H_3_BO_3_) were obtained from Aladdin Chemical Co., Shanghai, China. All the chemicals were in analytic grade and used without further purification.

### 2.2. Preparation

**Figure 1:**
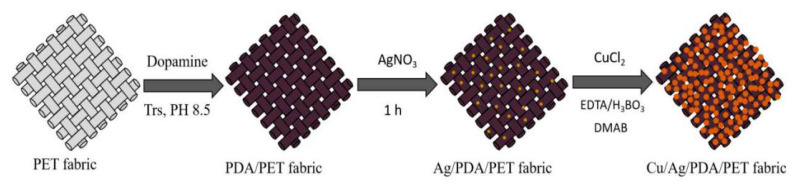
Schematic diagram of the synthesis process of Cu/Ag/polydopamine (PDA)/polyester (PET) fabrics.

The preparation process is illustrated in Figure 1. Firstly, polydopamine (PDA) was formed on the surface of PET fabrics through self-polymerization. Dopamine solution with a concentration of 10 mM was first dissolved in a Tris buffer solution. Then, the pH value of the solution was adjusted to 8.5 with HCl. PET fabrics were dipped into the freshly prepared dopamine solution at room temperature. After stirring for 24 h, the samples were washed thoroughly with DI water and dried in vacuum at 60 °C to obtain PDA/PET fabric.

Secondly, silver nanoparticles were deposited on the PDA-templated PET fabrics. The PDA/PET fabrics were added to Ag nitrate solution (30 mM) followed by stirring for 1 h in an overhead-shaker at room temperature. The samples were then washed thoroughly with deionized water and dried in vacuum at 60 °C. The product was denoted as Ag/PDA/PET fabrics.

Thirdly, Cu NPs were deposited on the Ag/PDA/PET fabrics through in situ reduction. The Cu plating aqueous solution was prepared by resolving 20 mL of CuCl_2_ (50 mM), 30 mL EDTA (50 mM) and 30 mL H_3_BO_3_ (100 mM) under stirring at room temperature. The pH value of the mixture solution was adjusted to 7.0 by adding 0.5 M NaOH at room temperature for 2 h. Under stirring, 20 mL DMAB solution (100 mM) was slowly dripped into the mixture solution. Then, the Ag/PDA/PET fabrics were dipped into the mixture solution for 2 h in an overhead-shaker at room temperature. Finally, the fabrics were washed with deionized water and dried in vacuum at 60 °C. The product was denoted as Cu/Ag/PDA/PET fabrics.

### 2.3. Characterization and Measurements

Scanning electron microscopy (SEM, JSM-5600LV, JEOL, Tokyo, Japan) with an energy dispersive X-ray spectroscopy (EDX, Oxford Instruments, Oxford, UK) was used to observe the surface morphology and detect elemental composition of the fabrics.

A transmission electron microscopy (TEM, 2100F, JEOL Inc., Tokyo, Japan) was used to observe the Cu NPs extracted from the coated fabrics by ultrasound.

Surface roughness of the fabrics was observed from an atomic force microscopy (AFM, NanoScope, Veeco, Santa Barbara, CA, USA) with the tapping mode in air.

X-ray photoelectron spectroscopy (XPS) measurement was performed on a PHI 5000C ESCA system with a Mg Ka source at 14.0 kV and 25 mA (Perkin-Elmer, Forster City, CA, USA).

The amount of Cu NPs coated on fabrics was measured by an inductively coupled plasma-mass spectrometry (ICP-MS, Agilent 7900, Agilent Technologies, Santa Clara, CA, USA).

The crystal structure of the fabrics was characterized by X-ray diffraction (XRD) on a Rigaku (Japan) D/max 2500 X-ray diffractometer using Cu K α radiation with the diffraction angle range 2θ = 10–80°, at 40 kV and 200 mA.

Thermogravimetry analysis (TGA) was conducted using a Netzsch TG209 F1 thermal analyzer (Selb, Germany) at a heating rate of 10 °C /min from 30 °C to 800 °C, in a nitrogen atmosphere (flow rate of 60 mL/min).

The antibacterial activity of Cu/Ag/PDA/PET fabrics was studied using colony counting method with *Escherichia coli* as the target bacteria. The test bacteria were cultivated at 37 °C in a yeast-dextrose broth containing 10 g/L peptone, 5 g/L sodium chloride, and 5 g/L yeast extract. For agar plates, 15 g/L agar was added to the broth and poured into petri dishes after sterilization in an autoclave. The *Escherichia coli* were transferred to a 2–3 mL broth solution for culturing at 37 °C under 250 rpm oscillation for 24 h. 250 μL of the diluted inoculum (containing 10^7^ cells) were transferred onto the fabrics (2.5 cm × 2.5 cm) in a sterile 250 mL wide-mouth glass jar. After incubation at 37 °C for 5 h, 100 mL sterile distilled water was added to each jar containing a fabric sample to elute bacteria. 200 μL of designated dilution of the elution with sterile water were placed on agar plates for incubation at 37 °C overnight. The bactericidal efficiency (Y) of the fabric samples was calculated by the following equation:

Y = (W_a_ − W_b_)/W_a_ (1)

where W_a_ and W_b_ are the average number of the colonies on the original PET fabrics and on the Cu NP-coated PET fabrics, respectively.

### 2.4. Electromagnetic Field Performance

The fabric samples were measured using N9917A FieldFox Microwave Analyzer (Agilent Technologies, Santa Clara, CA, USA) measurement system in the frequency range of 8.2–12.5 GHz (X-Band). The samples were cut into a rectangle with a dimension of 50 × 50 mm^2^ to fit the waveguide holder. The scattering parameters (S-parameters) were obtained from the Electromagnetic interference shielding effectiveness (EMI SE) test at room temperature. The total EMI SE can be calculated as follows [20,21]:

SE = −10log(P_T_/P_I_) (2)

where the EMI SE of a material is defined as the ratio of transmitted power (P_T_) to incident power (P_I_) of an EM wave.

## 3. Results and Discussion

### 3.1. Morphology

**Figure 2:**
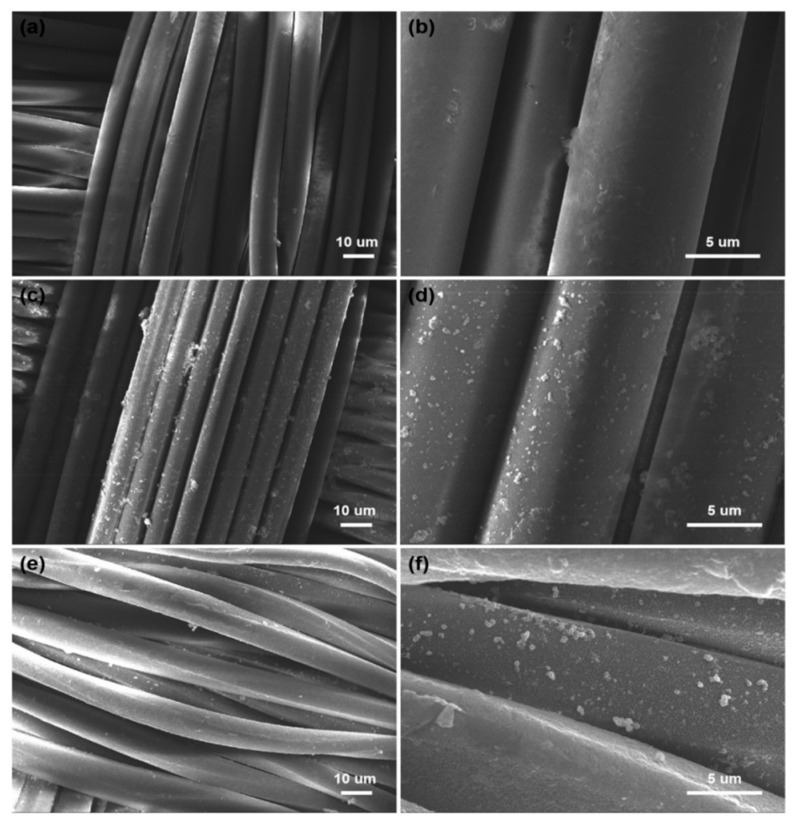
Scanning electron microscopy (SEM) images of (**a**,**b**) original PET fabrics; (**c**,**d**) PDA/PET fabrics and (**e**,**f**) Ag/PDA/PET fabrics.

The morphology PET fiber is relatively smooth and clean (Figure 2a,b). The surface of the PDA/PET fabrics becomes rough with many nanoparticles located on the surface of fibers. The change in morphology is mainly due to the formed PDA nanoparticles as a result of self-polymerization of dopamine under alkaline conditions (Figure 2c,d). As shown in Figure 2e,f the morphology of Ag/PDA/PET fabrics, the nanoparticles are uniformly deposited on the surface of fibers, indicating the successful reduction of Ag NPs on PDA/PET fabrics. PDA with catechol and amine functional groups has reduced Ag^+^ to Ag NPs via chelation.

**Figure 3:**
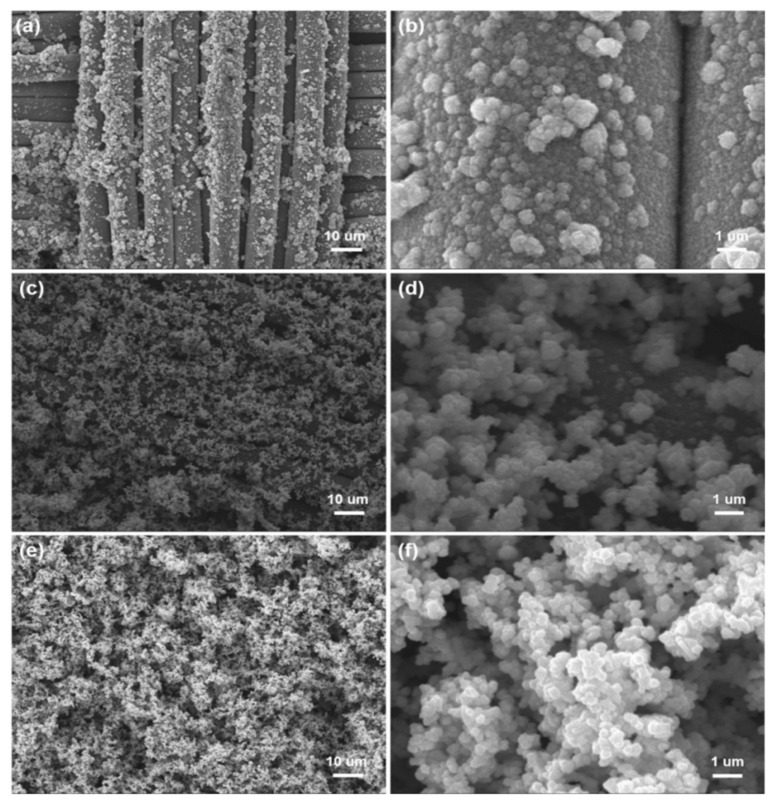
SEM images of Cu/Ag/PDA/PET fabrics with different magnifications: (**a**,**b**) reaction time of 2 h; (**c**,**d**) reaction time of 4 h; and (**e**,**f**) reaction time of 6 h.

For chemical plating, a surface without catalytic activity needs to be activated, so as to deposit metal particles with catalytic activity on the surface, such as palladium, gold, or silver. PDA contains many catechol and amine functional groups to reduce Ag^+^ into Ag NPs on fiber surface through chelation, and the Ag NPs as the catalytic activation seeds can catalyze chemical copper plating to deposit Cu NPs on the surface of fabrics [22,23,24]. In the initial stage of the reaction, copper ions in the copper plating solution were preferentially reacted with Ag NPs due to the strong catalytic activity of Ag NPs. As a result, Cu NPs were dispersed uniformly to cover the surface of fibers (Figure 3a). The fiber surface is covered by nanoparticles, but the profile is clearly visible (Figure 3b). Reaction time has a direct effect on the deposition of Cu NPs. When the reaction time was 4 h, copper ions were reduced to Cu NPs by the reducing agent with visible agglomeration on the surface of fibers (Figure 3c,d). As seen from Figure 3e,f, Cu NPs agglomerated seriously on the fiber surface when the reaction time was 6 h, and the fiber profile was hard to distinguish as a result. These results show that Ag NPs as the catalytic active centers promoted the deposition of Cu NPs on the surface of fibers with uniformity and density.

**Figure 4:**
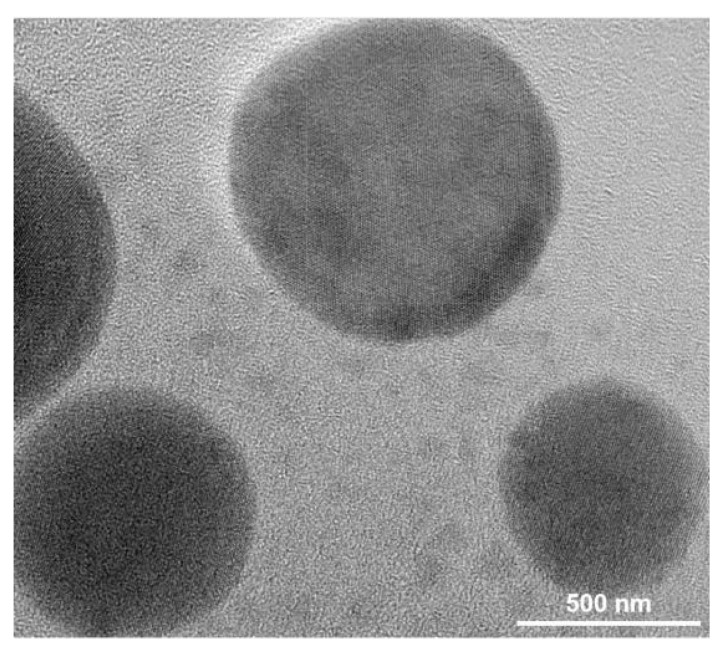
The transmission electron microscopy (TEM) image of the extracted Cu NPs from the Cu/Ag/PDA/PET fabric.

In order to determine the detailed size and morphology of the Cu NPs adhered to the fabrics, the nanoparticles were analyzed by TEM, as showed in Figure 4. Firstly, the nanoparticles-coated fabric was immersed in water and ultrasonic processed (30 min) to extract nanoparticles from the coated fabric. The solution was dispersed and dropped on a copper mesh for TEM test. The nanoparticles have a round shape and the size of the particles was about several hundred nanometers as seen from the TEM image.

**Figure 5:**
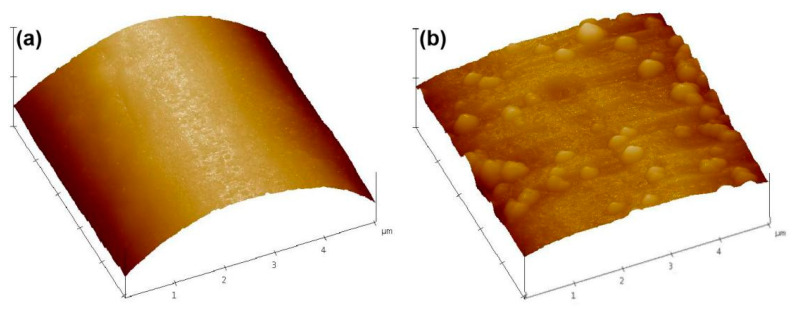
Atomic force microscopy (AFM) images of (**a**) original PET fiber; (**b**) Cu/Ag/PDA/PET fiber reaction time of 2 h.

To determine the effect of the Cu NPs on the surface roughness of the fiber, the surface roughness of the untreated and treated PET fiber samples were tested by AFM, as shown in Figure 5. The surface of pristine PET fiber is relatively smooth with a root mean square (RMS) value of 5.4 nm (Figure 5a). Whereas, after the deposition of Cu NPs, a significant increase in surface roughness was observed on the fiber surface (Figure 5b) and the RMS value augmented to 37.8 nm. The results of the AFM analysis indicate the presence of Cu NPs on the surface of the fiber.

### 3.2. Elemental Analysis

**Figure 6:**
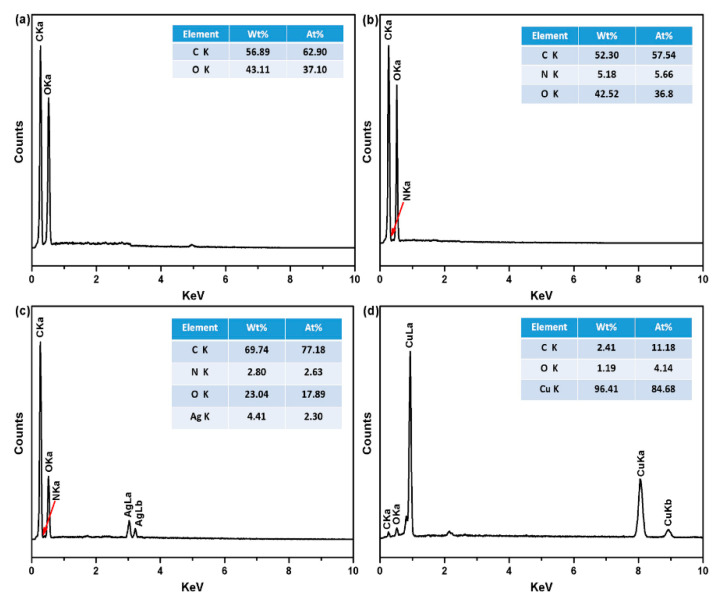
Energy dispersive X-ray spectroscopy (EDS) spectra of (**a**) original PET fabrics; (**b**)PDA/PET fabrics; (**c**) Ag/PDA/PET fabrics; and (**d**) Cu/Ag/PDA/PET fabrics.

Surface chemical elements of PET fabrics were determined by EDS spectroscopy, as depicted in Figure 6. Original PET fabrics contain only carbon (C) and oxygen (O) as the major elements (Figure 6a). The spectrum of the PDA/PET fabrics exhibits nitrogen (N) element with a weight percentage of 5.18% and atomic percentage of 5.66% (Figure 6b), and this is due to the N element from the structure of PDA. The presence of N element indicates the successful polymerization of PDA on the surface of PET fabrics [25]. After the deposition of Ag NPs, silver (Ag) element was detected at 3.0 keV with a content of 4.41 wt.% and atomic percentage of 2.30% (Figure 6c), confirming the presence of Ag NPs on the surface of fibers. There are three detected peaks corresponding to C, O, and Cu elements from the Cu/Ag/PDA/PET fabrics (Figure 6d), and the content of Cu elements is 96.41% and atomic percentage of 84.68%. N and Ag elements cannot be detected mainly because the surface of fibers is completely covered by Cu NPs.

**Figure 7:**
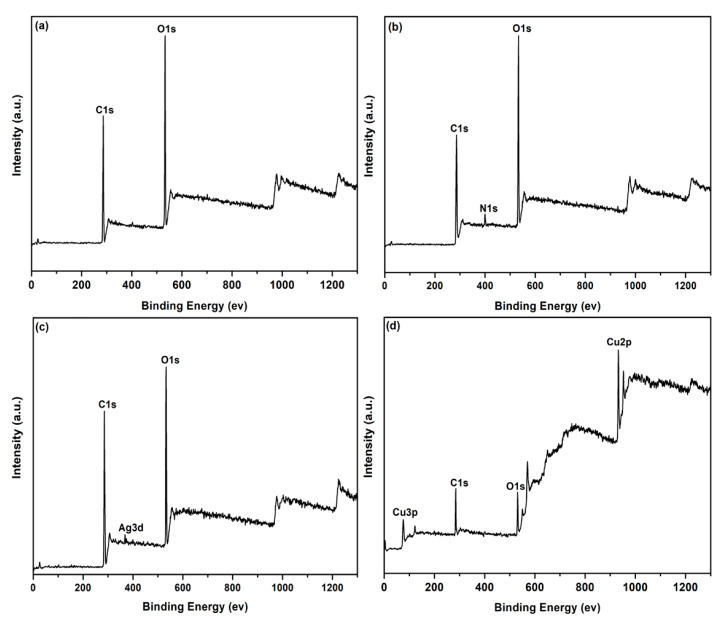
X-ray photoelectron spectroscopy (XPS) spectra of (**a**) original PET fabrics; (**b**)PDA/PET fabrics; (**c**) Ag/PDA/PET fabrics; and (**d**) Cu/Ag/PDA/PET fabrics.

The elemental compositions on the surface of the coated PET fabric was further investigated by XPS as shown in Figure 7. The XPS survey spectra reveal the presence of C1s and O1s signals for the original PET fabric (Figure 7a). Compared with the original fabric, a new N1s peaks can be detected for PDA/PET fabric (Figure 7b), which is attributed to the self-polymerization of PDA layer on the surface. Except for C1s, O1s, and N1s peaks, an extra Ag3d peak can be detected for Ag/PDA/PET fabric, indicating the presence of AgNPs on the surface of PET fabric (Figure 7c) [26]. For Cu/Ag/PDA/PET fabric (Figure 7d), the Cu 2p peaks have their major component at 932.6 eV (Cu 2p_3/2_) together with a satellite peak centered at 952.5 eV (Cu 2p_1/2_), which corresponds to metallic Cu [27].The strong signal of Cu 2p peak confirms the presence of Cu NPs on the surface of fabrics. These results are consistent with the EDS data.

### 3.3. XRD Pattern

**Figure 8:**
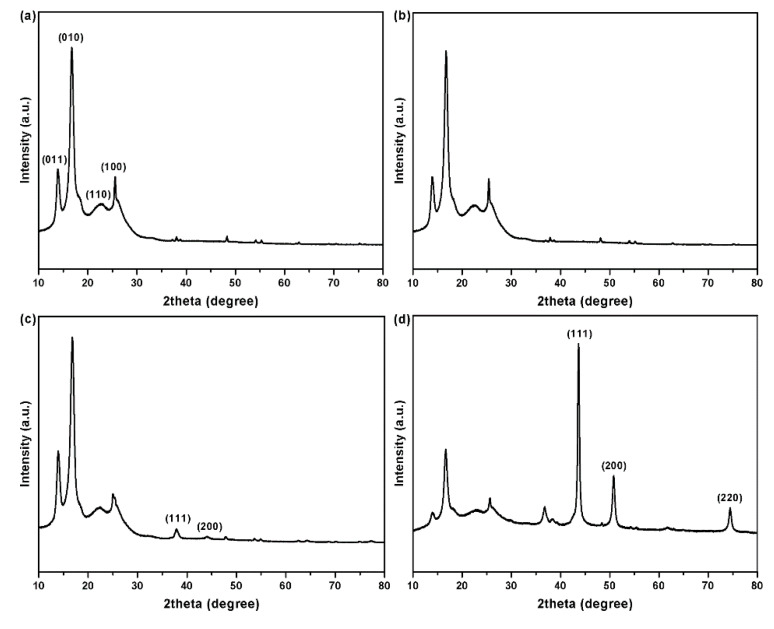
X-ray diffraction (XRD) patterns of (**a**) PET fabrics; (**b**) PDA/PET fabrics; (**c**) Ag/PDA/PET fabrics; and (**d**) Au/Ag/PDA/PET fabrics.

The XRD patterns of the PET fabrics before and after coating are shown in Figure 8. The original PET fabrics show four significant peaks at 14.0°, 17.5°, 22.7°, and 25.5° (Figure 8a), corresponding to four crystal planes of (011), (010), (110), and (100), respectively (Joint Committee on Powder Diffraction Standards, JCPDS card No. 00-050-2275) [28]. The PDA/PET fabrics show a similar XRD pattern, indicating that the PDA coating has little influence on the crystalline structure of PET fabrics (Figure 8b). This is mainly due to the amorphous structure of PDA formed on the surface of PET fabrics during the self-polymerization process [29]. Apart from the peaks of PET fabrics, the signals of Ag are observed in the diffraction pattern of the Ag/PDA/PET fabrics as shown in Figure 8c. There are two more characteristic peaks at 38.2° and 44.3° corresponding to the (111) and (200) planes of the face centered cubic crystal structure of Ag NPs (JCPDS card No. 65-2871) [30]. Due to the low content of silver, no other characteristic peaks can be detected. As shown in Figure 8d, the XRD pattern of the Cu/Ag/PDA/PET fabrics exhibits extra three remarkably sharpened diffraction peaks at 2θ = 43.5°, 50.7°, and 74.4° corresponding to (111), (200), and (220) crystal planes of metallic copper, respectively (JCPDS card No. 85-1326) [31]. The results further confirm that Cu NPs have been grown on the surface of PET fabrics.

### 3.4. Thermal Analysis

**Figure 9:**
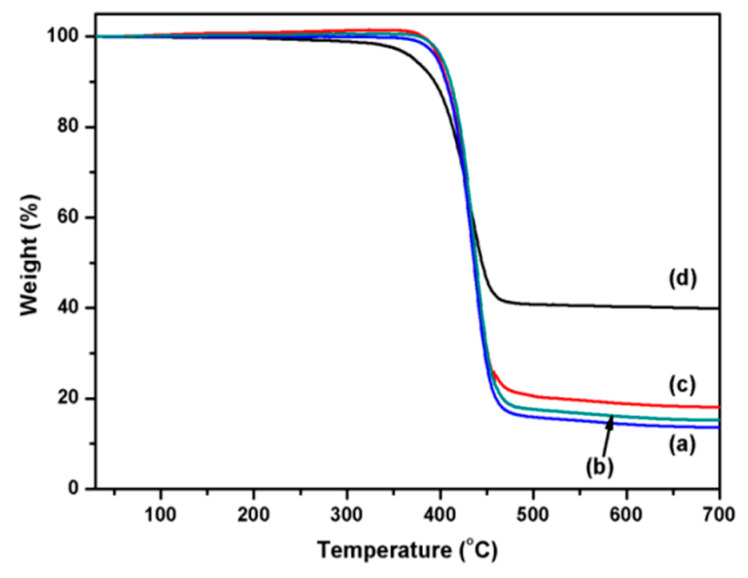
Thermogravimetry (TG) curves of (**a**) PET fabrics; (**b**) PDA/PET fabrics; (**c**) Ag/PDA/PET fabrics; and (**d**) Cu/Ag/PDA/PET fabrics.

The thermal stability of the coated PET fabrics was evaluated by thermogravimetric analysis. As shown in Figure 9, the thermogravimetry (TG) curves of the original PET and PDA/PET fabrics are almost the same (Figure 9a,b). The onset and endset decomposition temperature for original PET and PDA/PET fabrics are 406 and 455 °C with about 14.1% residue left at the end of the test (700 °C). The main reason is that the thickness of PDA on the fabric surface is only a few tens of nanometers, which has little influence on the fabrics during the thermal decomposition process. After the coating of Ag NPs, the onset and endset decomposition temperature are about 399 and 453 °C with about 18.1% residue left in the end (Figure 9c). It can be calculated that the Ag content is about 4%, which is consistent with the EDS analysis. The TG curve of the Cu/Ag/PDA/PET fabrics is shown in Figure 9d. The onset and endset decomposition temperature are 372 and 449 °C with 39.8% residue in the end. The main reason is that after copper plating the thermal conductivity of the copper is strengthened with accelerated decomposition, and the increased residue of coated polyester fabrics is ascribed to the immobilized Ag NPs and Cu NPs.

### 3.5. Durability

**Figure 10:**
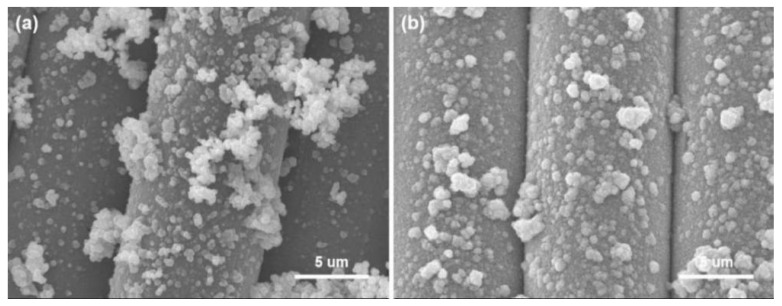
SEM images of Cu/Ag/PDA/PET fabrics before (**a**) and after (**b**) washing for 10 cycles.

Washing fastness is an important factor in practical applications, and the binding force between nanoparticles and fibers directly affects the durability of functional textiles. The Cu/Ag/PDA/PET fabrics were placed in a washing fastness tester, and the morphology of the fabrics was observed after 10 cycles of washing. As shown the SEM images in Figure 10, only a few Cu NPs were washed off from the fiber surface after washing, and the Cu NP film on surface of fibers has been maintained. The main reason is that PDA has strong adhesion for loading Cu NPs on the surface of fibers. Herein, PDA not only acts as a reducing agent for the reduction of silver ions to Ag NPs, but also as the adhesive layer for loading Cu NPs.

To examine the content of Cu NPs on the fabric surface, ICP-MS was used to analyze the elements of the coated fabric. The amount of Cu NPs changed from 1.84 to 1.65 mg/g after 10 cycles of washing. The result show that the Cu NPs has been well preserved on the fiber surface after washing, indicating the excellent durability of the coating, which can also be seen from the SEM images in Figure 10. The main reason is that PDA has strong adhesion for loading Cu NPs on the fiber surface.

### 3.6. Antibacterial Activity

**Figure 11:**
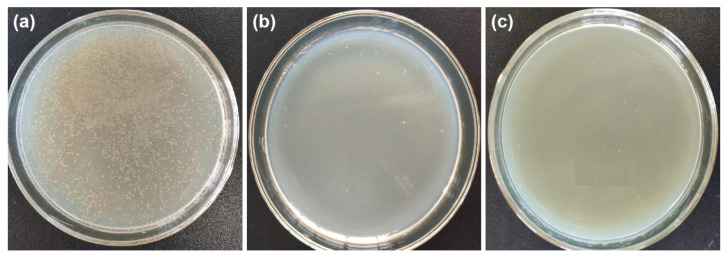
Antibacterial activities of (**a**) PET fabrics; (**b**) Cu/Ag/PDA/PET fabrics; and (**c**) after washing for 10 cycles.

Textile products are easy to breed bacteria. It is necessary to finish fabrics with antibacterial performance, and the most effective method for fabric antibacterial finishing is through attaching nanoparticles on the surface of fibers. However, nanoparticles are likely to aggregate and fall off, resulting in poor antibacterial properties. PDA as a template and Ag NPs as active catalyst to induce Cu NPs deposit uniformly on the surface of fabric. The antimicrobial activities of the original PET fabrics and Cu/Ag/PDA/PET fabrics were evaluated side-by-side using a colony count method, as shown in Figure 8. The petri dish was almost covered with bacteria (Figure 11a) indicates that the original PET fabrics have poor antibacterial properties. Compared with the original PET fabrics, only little bacteria exist on the culture dish for Cu/Ag/PDA/PET fabrics, and a 99.2% reduction has found in bacterial loading against *Escherichia coli* (Figure 11b). The highly enhanced antibacterial properties are attributed to the Cu NPs on the surface of fabrics that kill most of the adhered bacteria [32,33,34]. The coated fabrics also exist very few bacteria on the culture dish after washing, and a 98.7% reduction has found in bacterial loading against *Escherichia coli* (Figure 11c). Hence, the fact that washing has negligible effect on the antibacterial property, which is due to the vast majority of Cu NPs have been maintained on the fiber surface to prevent bacterial growth.

### 3.7. Electromagnetic Field Performance

**Figure 12:**
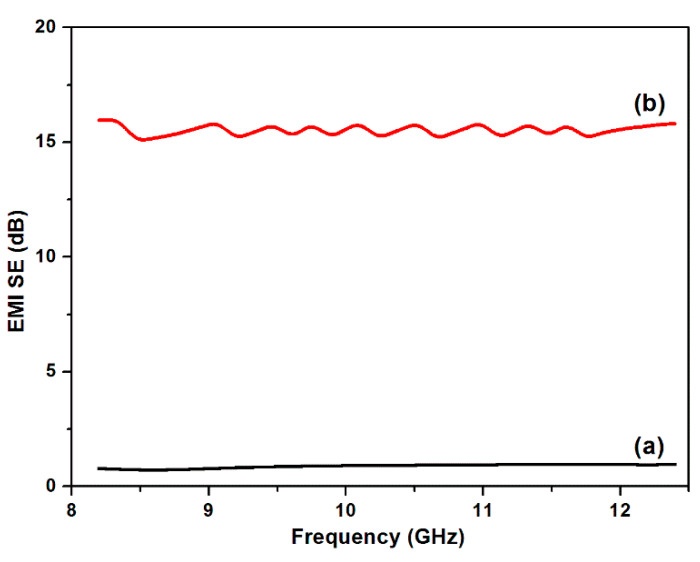
Electromagnetic interference shielding effectiveness (EMI SE) properties of (**a**) PET fabrics and (**b**) Cu/Ag/PDA/PET fabrics.

With the rapid development of electromagnetic technology, the increasing radiation from electromagnetic wave has raised great concerns in society due to its high risk to health. Functional textiles are widely used as electromagnetic shielding materials due to their light weight, flexibility, low cost and possibility of designing structure. Figure 12 shows the EMI SE of PET fabrics and Cu/Ag/PDA/PET fabrics in the X band frequency range from 8 to 12.5 GHz. The original PET fabric was almost transparent to electromagnetic waves due to its ultra-low electrical conductivity. As expected, the EMI SE increased to 15.5 dB after plating of Cu NPs on the PET fabric. Apparently, the ability to shield EM waves was dependent on the formation of an electrically conductive network on the fabric surface after plating of Cu NPs. In addition, the results show that the EMI SE value fluctuated with the frequency, which may be the irregular of conductive network formed on the PET fabric surface [35,36]. The Cu/Ag/PDA/PET fabrics exhibited good electromagnetic interference (EMI) shielding ability.

## 4. Conclusions

A simple but effective method was developed to immobilize Cu NPs on PET fabrics via catalyzing by Ag NPs on a PDA template. PDA as a reducing agent played a prominent role in the synthesis of Ag NPs on the PET fabric, serving as catalytic seeds for the subsequent in situ reduction of Cu NPs. Cu NPs were successfully deposited on PET fabrics as evidenced by morphology and crystalline structure together with TG analysis. The washing fastness test results showed that most Cu NPs were preserved even after 10 cycles of washing. The good durability is due to the strong adhesive ability of PDA and the binding between NPs and PDA. The results of antibacterial activities showed that the Cu NPs-coated PET fabrics have good antibacterial property against *Escherichia coli* with a 99.2% reduction in bacteria loading. The Cu NPs-coated PET fabrics also exhibit excellence electromagnetic interference (EMI) shielding ability with a maximum SE value of 15.5 dB. The coated PET fabrics with high performance antibacterial and EMI shielding properties have the potential to be applied as performance textiles.
